# Development and Verification of a Precolumn Derivatization LC-MS/MS Method for the Pharmacokinetic Study of Houttuynine of Houttuynia Essential Oil

**DOI:** 10.3390/molecules26082327

**Published:** 2021-04-16

**Authors:** Yuanyuan Liu, Yanfang Yang, Bangyuan Wang, Renyun Wang, Jianmei Pang, Yu Jiang, Yuling Liu

**Affiliations:** 1 State Key Laboratory of Bioactive Substance and Function of Natural Medicines, Institute of Materia Medica, Chinese Academy of Medical Sciences & Peking Union Medical College, Beijing 100050, China; liu20220883@163.com (Y.L.); yangyf@imm.ac.cn (Y.Y.); azqok2@163.com (B.W.); wry@imm.ac.cn (R.W.); pjmkerry@sina.com (J.P.); 17865562120@163.com (Y.J.); 2 Beijing Key laboratory of Drug Delivery Technology and Novel Formulation, Institute of Materia Medica, Chinese Academy of Medical Sciences & Peking Union Medical College, 1 Xiannongtan Street, Beijing 100050, China

**Keywords:** *Houttuynia cordata* Thunb., houttuynine, houttuynia essential oil, LC-MS/MS, pharmacokinetic study

## Abstract

Houttuynia essential oil (HEO) has excellent antiviral, anti-inflammatory, and other pharmacological effects, but the lack of effective analytical methods to quantify HEO in plasma has hindered its better clinical monitoring. Houttuynine (Hou) is one of the main active ingredients and quality control substances of HEO, so the pharmacokinetic study of HEO could be conducted by determining Hou blood concentration. Hou is active and not stable in plasma, which makes its blood concentration difficult to measure. In this work, a novel liquid chromatography tandem mass spectrometry (LC-MS/MS) method for Hou determination in rat blood was established that involves Hou being derivatized with 2, 4-dinitrophenylhydrazine to form a stable compound to prevent degradation. Herein, *p*-Tolualdehyde-2,4-dinitrophenylphenylhydrazone was selected as an internal standard substance and the LC-MS/MS method was evaluated for selectivity, precision, accuracy, calibration limit, matrix effect, recovery, and stability. Good linearity (r^2^ = 0.998) was reached in the range of 2–2000 ng/mL, and the lower limit of quantification of Hou was determined to be 2 ng/mL. The mean intra-assay accuracy ranged from 77.7% to 115.6%, whereas the intra-assay precision (relative standard deviation, RSD) was below 11.42%. The matrix effect value for Hou in rat plasma was greater than 75%, and for the internal standard (IS) it was 104.56% ± 3.62%. The extraction recovery of Hou were no less than 90%, and for the IS it was 96.50% ± 4.68%. Our method is sensitive and reliable and has been successfully applied to the pharmacokinetic analysis of Hou in rats given HEO via gavage and injection.

## 1. Introduction

*Houttuynia cordata* Thunb. belongs to the *Saururaceae* family and is a perennial herbaceous plant with a fishy smell. It is well known as both medicine and food in China, Korea, Japan, and Southeast Asia [1,2]. Its growing conditions tend to be shady and moist, and it is mainly distributed in southern areas such as in Guizhou and Wuhan in China [3]. *Houttuynia cordata* Thunb. has many pharmacological activities: anti-microbial, anti-inflammatory, anti-tumor, and so on [4,5,6,7]. It contains various compounds, including flavonoids, polysaccharides, alkaloids, volatiles, and fatty acids [8,9,10]. Among these, Houttuynia essential oil (HEO) is known to be the main active substance, which includes houttuynine (Hou), 2-undecanone, camphene, pinene, limonene, myrcene, and so on [11,12]. HEO has been widely used to treat tumors, osteoporosis, asthma, pneumonia, keratitis, and respiratory infections [13,14,15]. However, the lack of effective analytical methods to quantify HEO in plasma has hindered its better clinical monitoring. Since Hou is one of the main active ingredients [16,17] and quality control materials in HEO, the pharmacokinetic study of HEO could be conducted by determining the concentration of Hou in blood.

Gas chromatography-mass spectrometry (GC-MS), solid-phase microextraction (SPME)-GC-MS, high-performance liquid chromatography (HPLC), and liquid chromatography tandem mass spectrometry (LC-MS/MS) are widely used for the detection of volatile oil content [2,3,18,19,20,21,22]. At present, gas chromatography (GC) is the most widely used for Hou determination [2,3], but it cannot detect Hou in blood. Moreover, Hou is a *β*-dicarbonyl compound with chemical activity, which is easily degraded, polymerized, oxidized [23], and not stable in plasma, making its blood concentration difficult to measure. Therefore, establishing an efficient and accurate method to quantify Hou in plasma is key to conducting pharmacokinetic studies of HEO.

**Figure 1:**
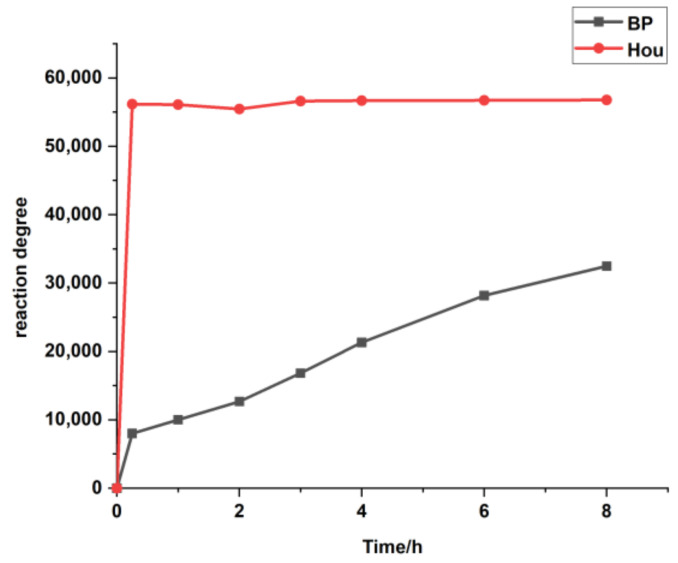
The response values of the derivatives in the LC-MS/MS with increasing reaction time. BP: benzophenone: Hou: houttuynine.

Based on the determination method of sodium houttuynia (SH, an adducted compound of sodium bisulfite and Hou) in human plasma reported by Duan [18], Hou was derived with 2,4-dinitrophenyl hydrazine (DNPH) to form a stable compound to prevent its degradation, and the content of Hou was determined by LC-MS/MS using the internal standard (IS) method. However, we found that the method was not suitable for monitoring the changes of Hou in vivo. First, the derivatization efficiencies of the IS (benzophenone, BP) and Hou are very different. As can be seen from Figure 1, Hou reacts completely within 15 min, while the reaction of the same amount of BP is still incomplete after 8 h. This phenomenon greatly reduced the accuracy of the Hou determination. Second, it requires the use of 20% sulfuric acid, which is corrosive and rather unsafe. Third, the pre-processing steps are complex. Therefore, it is of great importance to find an appropriate IS, optimize the plasma treatment method, and establish an efficient LC-MS/MS qualitative detection method for Hou to pave the way for the clinical application of *Houttuynia cordata* Thunb.

In this study, the hydrolyzed product of SH, Hou, was isolated and purified. GC and nuclear magnetic resonance (NMR) were used to determine its purity and structure. *p*-Tolualdehyde-2,4-dinitrophenylhydrazone (PTD), which does not participate in the derivatization reaction, was selected as an IS to improve the accuracy of Hou detection. To improve the safety of the method, 2% hydrochloric acid (HCl) was chosen as a catalyst. Acetonitrile was used as a solvent to remove protein in plasma samples by centrifugation. Thus, a novel LC-MS/MS method was constructed to determine Hou levels in biological samples.

In addition, HEO has multiple components and can play a role in multiple targets; compared with a single component such as Hou or methyl nonone, it is more likely to be used as a drug. However, HEO obtained by the traditional extraction process was easily destroyed by high temperatures [24], which greatly reduces its efficacy and increases the toxic side effects such as hemolysis. Especially when given by injection, HEO can cause frequent hemolysis [25]. This research group previously used low-temperature extraction technology to prepare HEO with a 55% Hou content, and the safety evaluation confirmed that the safety of low-temperature extraction of HEO is much greater than that of steam distillation at the same concentration. Therefore, HEO with a 55% Hou content was chosen as the object in the study, and the in vivo pharmacokinetics after oral administration and injection were evaluated based on the determination of Hou blood concentration using the established LC-MS/MS method.

## 2. Results

### 2.1. Method Development

**Figure 2:**
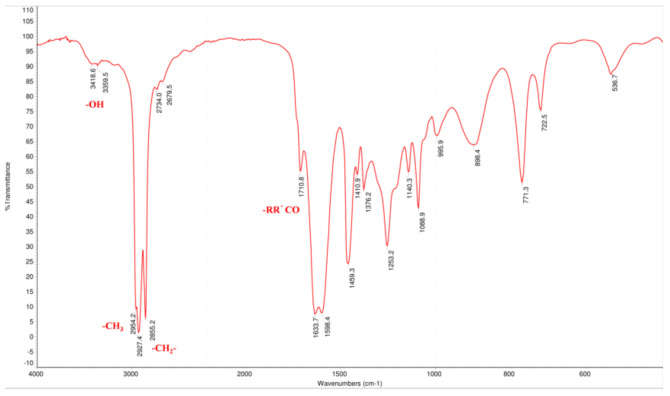
The IR spectrum of Hou.

Hou standard was successfully prepared by hydrolyzing SH. Its structure and purity were determined based on GC, electron ionization mass spectrometry (ESI-MS), IR (infrared spectroscopy), NMR (nuclear magnetic resonance), and GC data. The ESI-MS showed the molecular ion peaks at *m/z* 198.1 [M]^+^, this result is in accordance with previous findings [26]. The IR spectrum (Figure 2) exhibited absorption bands associated with conjugated carbonyl (1633.6, 1598.4 cm^−1^), hydroxyl (3418.6 cm^−1^), methyl (2954.2 cm^−1^), and methylene (2927.4 cm^−1^), which suggested that Hou has an enol structure.

**Figure 3:**
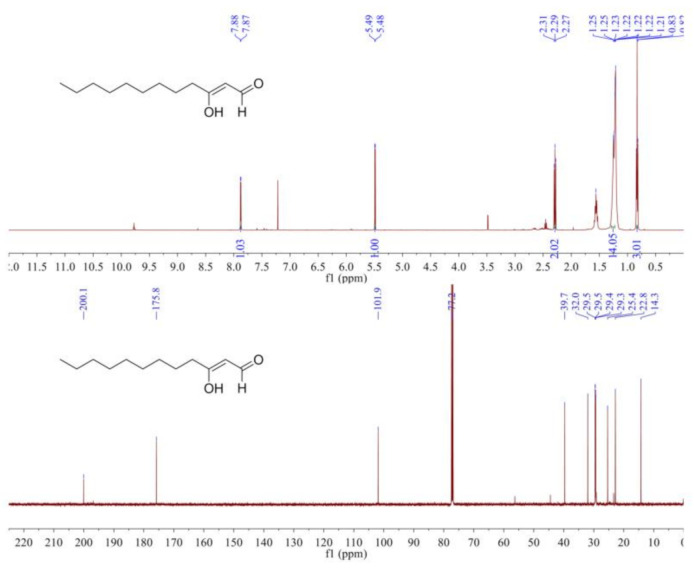
The ^1^H NMR spectrum and ^13^C NMR spectrum of Hou in CDCl_3_.

The ^1^H NMR spectrum (Figure 3) indicated an aldehyde hydrogen at δ_H_ 7.88 (1H, d, *J* = 4.0 Hz), a conjugated enol hydrogen at δ_H_ 5.49 (1H, d, *J* = 4.0 Hz), a methyl signal at δ_H_ 0.83 (3H, t, *J* = 7.0 Hz), and eight methylene signals at δ_H_ 2.31–1.21. The ^13^C NMR spectrum (Figure 3) showed 12 carbon signals, which included one aldehyde carbon signal (δ_C_ 200.1), two double-bond carbon signals (δ_C_ 175.8, 101.9), one methyl carbon signal (δ_C_ 14.3), and eight methylene carbon signals. From the above data, we inferred that the structure of the compound was 3-oxo-dodecanal, also known as Hou.

**Figure 4:**
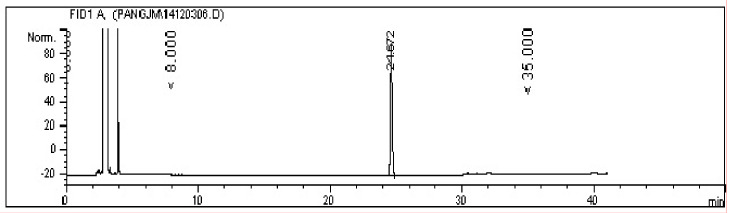
The GC spectrum of Hou.

The GC spectrum in Figure 4 suggests that the prepared Hou standard was pure, in which the impurity peak was lower than the peak of Hou (retention time, 24.672 min), and its relative content was 98.2% as calculated by the area normalization method.

**Figure 5:**
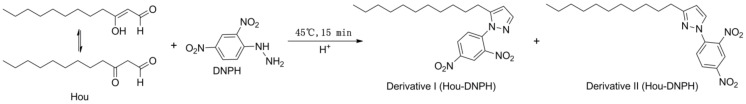
The equation of the Hou and 2,4-dinitrophenyl hydrazine (DNPH) reaction.

We successfully obtained HEO with a 55% Hou content according to the reported method [27]. DNPH, a reaction reagent widely used for the determination of carbonyl compounds [28,29,30] was selected as a derivative reagent for Hou. PTD is a stable product of formaldehyde and DNPH, and it has a molecular weight and structural characteristics similar to those of Hou-DNPH derivatives. Therefore, PTD was used as an IS to improve the detection accuracy. The equation of the Hou and DNPH reaction is shown in Figure 5.

HCl (at concentrations of 1–20%) and formic acid (FA) were used in the same concentration range as the catalysts for derivatization; the results are shown in Table 1 and Table 2, respectively. It can be seen from Table 1 that HCl significantly improved the derivatization efficiency and yield of Hou when compared to those of FA. The results for different concentrations of HCl shown in Table 2 suggest that 2% HCl obviously improved the derivatization efficiency.

### 2.2. Method Validation

#### 2.2.1. Selectivity

**Figure 6:**
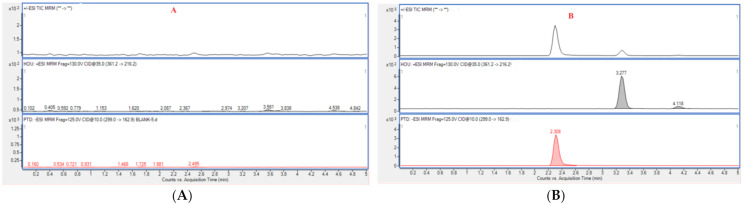

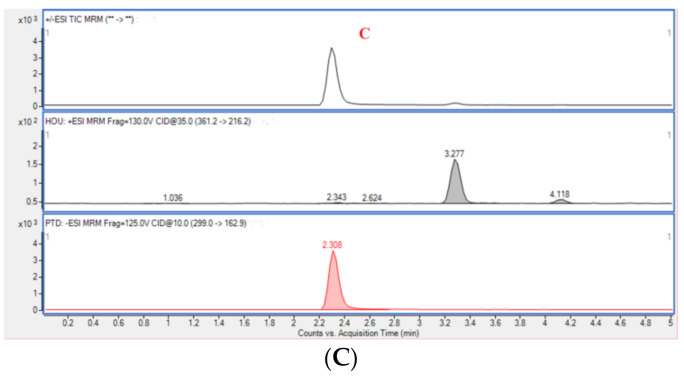
Typical multiple reaction monitoring (MRM) chromatograms of blank rat plasma (**A**), blank plasma sample spiked with analytes (**B**), and a plasma sample from a Houttuynia essential oil (HEO)-treated rat with *p*-Tolualdehyde-2,4-dinitrophenylhydrazone (PTD) (**C**).

The results of the selectivity evaluation indicated that there was no obvious interference signal detected at the retention times of Hou (3.288 min) and PTD (2.308 min) for blank plasma. Typical multiple reaction monitoring (MRM) chromatograms of blank rat plasma, plasma containing Hou and PTD, and plasma from a rat administrated with HEO with PTD are displayed in Figure 6.

#### 2.2.2. Calibration Curve and Lower Limit of Quantification (LLOQ)

The standard curve studied in the range of 2–2000 ng/mL showed good linearity, and can be specifically described as Y = 0.010X − 0.003472 (*n* = 6, r^2^ = 0.998), where X is the plasma concentration of Hou and Y is the ratio of the peak area of Hou to that of the IS. The lower limit of quantification (LLOQ) of Hou was determined to be 2 ng/mL (was set at 2 ng/mL), which is sufficient for pharmacokinetic analysis in rat plasma.

#### 2.2.3. Accuracy and Precision

Intra-assay precision and accuracy results (n = 6) for low, medium, and high concentration levels are reported in Table 3. The mean intra-assay accuracies ranged from 77.7% to 115.6%, whereas the intra-assay precision, described as the relative standard deviation (RSD) of the calculated concentrations, was below 11.42%. These results demonstrated the method for quantitative determination of Hou in rat plasma was reliable and repeatable.

#### 2.2.4. Matrix Effects and Recovery

The matrix effect values for Hou at 10 ng/mL, 50 ng/mL, 200 ng/mL, and 2000 ng/mL were 98.44% ± 13.21%, 76.58% ± 8.17%, 86.84% ± 8.74%, and 94.32% ± 10.69%, respectively; the matrix effect for the IS at 33.33 μg/mL was 104.56% ± 3.62%, which indicated that the co-eluting endogenous substances did not influence the ionization of the analytes. The extraction recoveries of Hou at 10 ng/mL, 50 ng/mL, 200 ng/mL, and 2000 ng/mL in rat plasma were 91.32% ± 20.75%, 94.11% ± 8.70%, 110.70% ± 13.30%, and 95.38 ± 17.61%, respectively, with RSD% values below 22.72%, and the recovery of the IS was 96.50% ± 4.68%, with an RSD% value of 4.85%, which indicated that the method established to treat plasma samples has no effect on determination results and is feasible.

#### 2.2.5. Stability

The stability was evaluated at low, medium, and high concentration levels (*n* = 6) and the results are presented in Table 4. The data demonstrate that the Hou derivatives are stable for 8 h in plasma at room temperature (25 °C).

Taken together, the results demonstrate that the LC-MS/MS method established in this work is reliable, sensitive, and reproducible and can be used for pharmacokinetic analysis of Hou in rat plasma.

### 2.3. Pharmacokinetic Study

**Figure 7:**
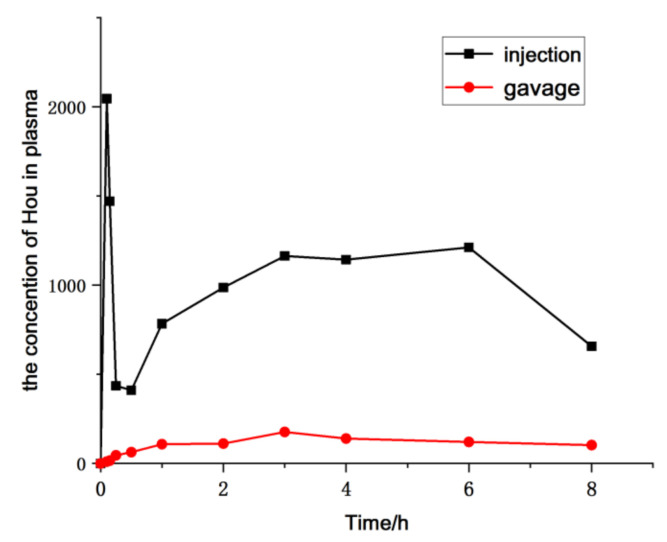
Mean plasma concentration–time profile of Hou in rats after a single administration of HEO by different administration forms (*n* = 6).

The pharmacokinetics of HEO administrated to rat via gavage and injection were evaluated based on the determination of Hou blood concentration by the established LC-MS/MS approach. Furthermore, the parameters of the pharmacokinetics were calculated by a non-compartmental model using the Drug and Statistics Software (DAS), version 3.2.8. Mean plasma concentration–time profiles are exhibited in Figure 7, and the key pharmacokinetic parameters are calculated and listed in Table 5. The results showed that the maximum plasma concentration (*C*_max_) of Hou was 222.48 ng/mL, the AUC was 2451.837 μg/L × h, the absolute bioavailability was 7.08% in oral administration, which also suggested that HEO can be absorbed orally to some extent, but its absolute bioavailability is lower than that of the injectable drug.

### 2.4. Safety Evaluation

The results showed that the animals in the intravenous administration group had reduced activity, lassitude, black tail, and hemolysis in blood samples, while there was no abnormal condition and no hemolysis in blood samples in the oral administration group. Those results indicate that the oral administration is safer and has potential as a drug delivery method. Therefore, the urgent problem to be solved in future research is to develop an appropriate new delivery system to improve the efficacy of HEO and improve the bioavailability of Hou.

## 3. Materials and Methods

### 3.1. Materials

*H. cordata* Thunb was provided by Yichang Jiahao Ecological Agriculture Development Co., Ltd. (Wuhan, China). Sodium houttuynia was obtained from Beijing Saige Bio-Pharmaceutical Co., Ltd. (Beijing, China). DNPH and PTD were obtained from Aladdin Reagent (Shanghai) Co., Ltd. (Shanghai, China). Heparin sodium was provided from Wuhan Yuancheng Co-created Technology Co., Ltd. (Wuhan, China). Acetonitrile and MS-grade FA were purchased from Thermo Fisher Scientific (Fair Lawn, NJ, USA).

Sprague-Dawley (SD) male rats (200 ± 30 g) were supplied by Beijing HFK Biotechnology Co. Ltd. (Beijing, China). The animal experiments met the rules set by the Ethics Committee of the Experimental Animal Center of Beijing E-Zhuang Biomedical Park, and the approval number is 2019S019.

### 3.2. Preparation of the Hou Standard

Hou was prepared by hydrolyzing SH in a 0.01 M Na_2_CO_3_ solution. In short, SH (2.0 g) was weighted precisely, transferred into a conical flask, and dissolved in a 0.01 M Na_2_CO_3_ solution. The system was stirred in an ice water bath for 15 min and was then extracted with dichloromethane. After vacuum drying (Gardner Denver, Karatasstr, Germany), Hou was obtained and was stored at −80 °C for further utilization and characterization.

### 3.3. Preparation of HEO

HEO was extracted and determined according to the reported method in reference [26]. Briefly, fresh Houttuynia cordata was cut into 1 cm lengths, soaked with ethyl acetate (the ratio of solid to liquid was 1:1.5), extracted by ultrasonication for 30 min, sealed, placed in a sunless place for 3 days, and concentrated to obtain crude HEO. After ethanol sinking, *n*-hexane extraction, and macroporous resin column chromatography, the HEO for pharmacokinetic and pharmacodynamic studies was obtained and analyzed by an Agilent 6890N series GC system with an Agilent DB-5 capillary column GC (30 m × 0.25 mm, 0.25 µm, Agilent Technologies, Santa Clara, CA, USA) for separation, and a hydrogen flame ionization detector (FID) was used for quantitative analysis [16].

### 3.4. LC-MS/MS Instrumentation and Conditions

We used a 6410B triple quadrupole LC-MS/MS system (Agilent, MA, USA) consisting of an Agilent 1200 RRLC system (Agilent Co.) coupled to a triple quadrupole MS analyzer with an ESI source. For chromatographic separations, we used an Eclipse XDB-C18 column (150 × 3.0 mm, 5 µm) (Agilent Technologies, Santa Clara, CA, USA). The mobile phase, consisting of acetonitrile/water/FA (90:10:0.1, *v*/*v*/*v*), was used in the gradient elution procedure at a flow rate of 0.5 mL/min. Ten-microliter aliquots of the sample solution were injected for analysis. The separation run was finished within 5 min of sample injection at 25 °C.

Quantification was performed in an MRM mode using ESI in the negative ion mode: for PTD, IS, *m/z* 299–*m/z* 162.9 (fragmentor, 130 V; collision energy, 35 eV); the optimized ion mode for Hou-DNPH was *m/z* 361.2–*m/z* 216.2 (fragmentor, 125 V; collision energy, 10 eV). The optimum operating parameters were as follows: drying gas temperature, 300 °C; drying gas flow, 8 L/min; nebulizer pressure, 45 psi; and capillary voltage, 3.55 kV. Data were acquired and processed using the Mass Hunter workstation (Agilent).

### 3.5. Preparation of Stock Solutions and Calibration Standards

PTD was used as an IS for the analyses, and BP was used as another IS for comparison. Hou and BP-Hou were dissolved in acetonitrile to form stock solutions of 1 mg/mL. Then, working solutions of 12–12,000 ng/mL were prepared from the stock solution and stored at 4 °C.

A certain amount of PTD was diluted in acetonitrile under ultrasonication to obtain working solutions of 200 μg/mL and 100 μg/mL. The derivative reagent, DNPH, was prepared by dissolving DNPH in acetonitrile containing 2% hydrochloric acid followed by ultrasonication to obtain a working solution of 2 mg/mL.

### 3.6. Plasma Sample Pretreatment

Blank rat plasma (100 μL), PTD (200 μg/mL), Hou working solution (50 μL), and DNPH solution (100 μL) were added to Eppendorf (EP) tubes (1.5 mL), vortexed for a few minutes, and shaken in a 45 °C water bath oscillator for 15 min. Then, the system was centrifuged at 14,000 rpm, and the supernatant was removed. The above centrifugation was repeated, and the plasma samples of Hou were obtained.

### 3.7. Method Validation

A full validation of the method was performed in accordance with the Guidance for Industry Bioanalytical Method Validation [31]. The validation method was conducted for Hou in plasma according to the validation rules for biological analysis methods developed by the Food and Drug Administration (FDA). This analytical method was validated based on specificity, linearity, precision, accuracy, recovery, and stability.

#### 3.7.1. Selectivity

The selectivity of the method was conducted by comparing the blank plasma samples to plasma containing Hou and PTD, and plasma from HEO-treated rats with PTD. Finally, whether the endogenous substances from blank plasma interfered the analytes or not was also studied.

#### 3.7.2. Calibration Curve

Calibration curves of Hou were performed from 2 to 2000 ng/mL. Linearity was determined by plotting the ratio of the peak area of the analytes to that of PTD (IS) versus the analyte concentrations and applying least-squares linear regression. A correlation coefficient (r^2^) higher than 0.99 was identified as linearity.

#### 3.7.3. Precision and Accuracy

The intra-assay precision and accuracy were tested. In this work, quality control (QC) samples of plasma at high, middle, and low concentrations (six parallels at each concentration) on a single day were studied. The variability of determination was expressed as the RSD%, and the accuracy was expressed as the relative error (RE%). Herein, the accuracy data within ± 15% RE from the nominal values and a precision data within ± 15% RSD were acceptable. However, the precision and accuracy data of the LLOQ should be within ± 20%.

#### 3.7.4. Matrix Effect and Extraction Recovery

The matrix effect was determined at three QC concentrations by comparing peak responses of post-extraction blank plasma (n = 6) spiked with QC samples with those of neat standard solutions. The extraction recovery was measured by comparing the responses of analytes obtained from extracted samples with blank plasma extracts spiked with QC samples. Meanwhile, the matrix effect and extraction recovery of the IS with a single concentration were also investigated.

#### 3.7.5. Stability

The stability of Hou-DNPH in plasma at high, middle, and low concentrations (*n* = 6 for each concentration) was investigated by keeping the samples at room temperature (25 °C) for 8 h.

### 3.8. Pharmacokinetic Study

#### 3.8.1. Animal Study

Twelve male SD rats (200 ± 30 g) were kept in an appropriate environment for three days before the start of the experiment. Before drug administration, the rats were fasted except for free water access for 12 h. All animals were randomly divided into two groups with six animals in each group. Blood samples (0.2 mL) were withdrawn at specific time intervals (0 h, 5 min, 10 min, 15 min, 30 min, 1 h, 2 h, 3 h, 4 h, 6 h, 8 h, 12 h, and 24 h) after administration in heparinized tubes. The samples were immediately centrifuged at 3000 rpm, and kept at −20 °C in a refrigerator.

#### 3.8.2. Plasma Sample Preparation

Plasma samples (100 μL) were mixed with 100 μL of DNPH (2 mg/mL), 50 μL of IS (PTD, 200 µg/mL), and 150 μL acetonitrile (containing 2% HCl) with uniform mixing, followed by vigorous vortexing for 3 min to precipitate the protein. Then, all samples were shaken in a water bath oscillator for 15 min at 45 °C. The solutions were then centrifuged. The centrifugation step was repeated once, and plasma samples of Hou were obtained and subjected to LC-MS/MS analysis.

#### 3.8.3. Safety Evaluation

The safety evaluation was conducted by observing the living status and hemolysis of the above pharmacokinetic experimental animals after administration.

#### 3.8.4. Data Analysis

Pharmacokinetic parameters including AUC (0–t), AUC (0–∞), MRT (0–t), MRT (0–∞), CL_z_ and so on, were calculated using the DAS (version 3.2.8, Chinese Pharmacological Association, Beijing, China). The *C*_max_ was obtained from the concentration–time curve. The area under the plasma concentration–time curve from zero to the time of the last measurable sample AUC (0–t)), area under the plasma concentration–time curve from zero to infinity AUC (0−∞), mean residence time from zero to the time of the last measurable sample (MRT_0−t_), mean residence time from zero to the time of infinity (MRT_0−__∞_), and CL_z_ were calculated using the DAS.

## 4. Conclusions

We developed a novel LC-MS/MS method for Hou determination in biological samples. The method involves derivatization with DNPH. PTD is used as an IS and 2% HCl as a catalyst. The method was demonstrated to be selective, linear, precise, and accurate for the determination of Hou. Good linearity (r^2^ = 0.998) was reached in the range of 2–2000 ng/mL, and the LLOQ of Hou was determined to be 2 ng/mL. The mean intra-assay accuracy ranged from 77.7% to 115.6%, whereas the intra-assay precision (RSD) was below 11.42%. The matrix effect value for Hou in rat plasma was greater than 75%, and for the IS it was 104.56% ± 3.62%. The extraction recovery of Hou was no less than 90%, and for the IS it was 96.50% ± 4.68%. Our new method was successfully applied to the pharmacokinetic study of HEO administrated to rats via gavage and injection. The results showed that the HEO can be absorbed orally to some extent, but the absolute bioavailability is lower than that of the injectable drug. The safety results of the two administration modes suggested that oral administration is safer and has the potential for drug development. Based on the results, an appropriate new delivery system should be developed for maximizing the efficacy of HEO and thus improving the bioavailability of Hou.

## Figures and Tables

**Table 1:** Comparison of derivative results of HCl and formic acid (FA) at different concentrations. IS: internal standard.

Catalyst	Concentration (%)	Response Values * of the IS	Response Values * of Hou-Derivatives
HCl	20	9628	235,556
HCl	10	12,158	244,090
HCl	5	14,738	243,659
FA	20	49,170	52,187
FA	10	59,359	22,483
FA	5	61,493	9440

* response values mean the response strength of the compound on the mass spectrum.

**Table 2:** Influence of different HCl concentrations on the results of derivatization.

Catalyst	Concentration (%)	Response Values * of the IS	Response Values * of Hou-Derivatives
HCl	5	7867	379,525
HCl	5	11,956	348,219
HCl	2	10,764	423,701
HCl	2	10,146	419,683
HCl	1	15,631	23,714
HCl	1	20,048	52,315

* response values mean the response strength of the compound on the mass spectrum.

**Table 3:** Accuracy and precision results of Hou derivatives (*n* = 6). RSD: relative standard deviation.

Analyte	Concentration (ng/mL)	Intraday Stability		
		Measurement (ng/mL)	Accuracy (%)	RSD (%)
**Hou-Derivatives**	10	11.56 ± 1.32	115.6	11.42
	50	38.84 ± 2.04	77.7	5.25
	200	189.33 ± 21.02	94.7	11.10

**Table 4:** Stability results of Hou derivatives in plasma (*n* = 6).

Analyte	Concentration (ng/mL)	Intraday Stability		
		Measurement (ng/mL)	Accuracy (%)	RSD (%)
**Hou-Derivatives**	10	8.98 ± 0.71	89.8	7.85
	50	45.87 ± 1.46	91.7	3.18
	200	172.16 ± 10.19	86.1	5.92

**Table 5:** Blood concentration of Hou after a single administration of HEO by different administration forms (*n* = 6).

Parameter	Unit	Injection	Gavage
*T*_1/2_	h	5.987 ± 0.52	6.45 ± 0.94
*T*_max_	h	0.033 ± 0.012	3.71 ± 1.07
*C*_max_	μg/L	2010 ± 350	222.48 ± 20.38
AUC(0–t)	μg/L × h	32,693.2 ± 1320	2451.79 ± 300.01
AUC(0–∞)	μg/L × h	34,630.466 ± 1436	2451.837 ± 432.74
MRT(0–t)	h	7.575 ± 1.21	8.914 ± 2.73
MRT(0–t)	h	9.153 ± 0.98	11.594 ± 1.85
*V*_z_	L/kg	5.611 ± 0.97	85.81 ± 5.74
CLz	L/h·kg	0.638 ± 0.086	8.856 ± 3.68
*F*	%	100	7.08

## Data Availability

The data presented in this study are available on requested from the authors.

## Sample Availability

Samples in this study can be checked with the author, Yuanyuan Liu.
